# Supplementary figures and images for: Positive Effects on Emotional Stress and Sleep Quality of Forest Healing Program for Exhausted Medical Workers during the COVID-19 Outbreak
Source: Int J Environ Res Public Health. 2022 Mar 7;19(5):3130. doi: 10.3390/ijerph19053130 (PMC8910265; doi:10.3390/ijerph19053130)

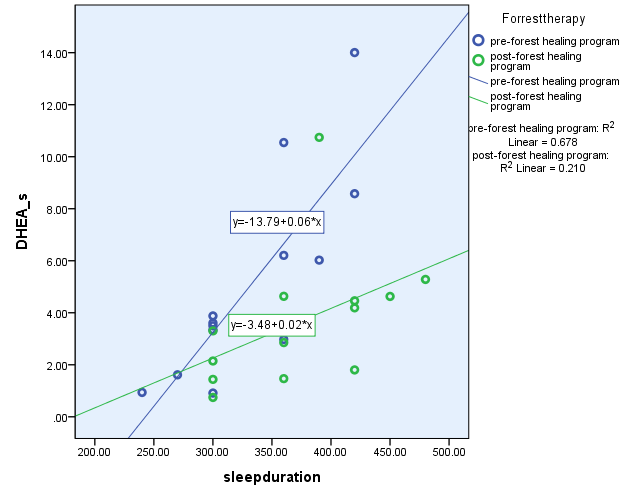

Supplement: Supplementary file 1 [file ijerph-19-03130-s001.zip › Figure S1.png]

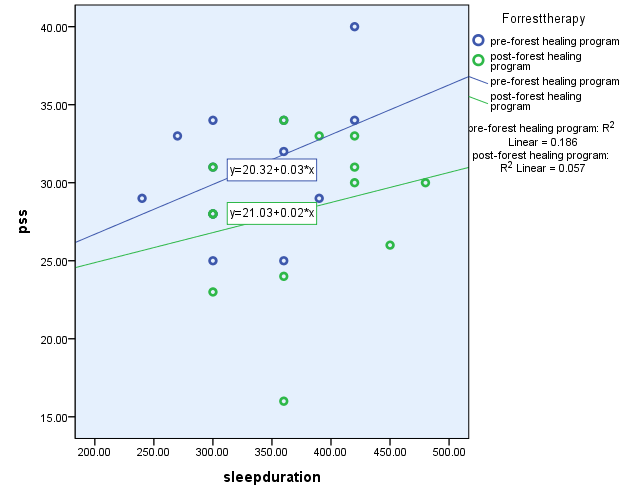

Supplement: Supplementary file 1 [file ijerph-19-03130-s001.zip › Figure S2.png]
